# Efficacy of Repeated Applications of Bacteriophages on *Salmonella enterica*-Infected Alfalfa Sprouts during Germination

**DOI:** 10.3390/pathogens11101156

**Published:** 2022-10-06

**Authors:** Catherine W. Y. Wong, Siyun Wang

**Affiliations:** Food, Nutrition and Health, University of British Columbia, 120-2205 East Mall, Vancouver, BC V6R 1Z4, Canada

**Keywords:** *Salmonella enterica*, alfalfa sprouts, bacteriophage, repeated applications

## Abstract

Nontyphoidal *Salmonella enterica* is one of the leading pathogens for foodborne outbreaks in a multitude of food commodities, including alfalfa sprouts, which are commonly consumed raw. The food industry has commonly used chlorinated washes, but such methods may not be perceived as natural; this can be a detriment as a large portion of sprouts are designated for the organic market. A natural and affordable antimicrobial method that has been acquiring popularity is the use of bacteriophages. This study compared the efficacy of repeated daily applications and a single application of two separate bacteriophage cocktails (SE14, SE20, SF6 and SE14, SF5, SF6) against four *Salmonella enterica* (*S. enterica*) strains on germinating alfalfa sprout seeds from days 0 to 7. The results show *S.* Enteritidis to be the most susceptible to both cocktails with ~2.5 log CFU/mL decrease on day 0 with cocktail SE14, SF5, and SF6. *S. enterica* populations on all strains continued to grow even with repeated daily bacteriophage applications but in a significantly decreased rate (*p* < 0.05) compared with a single bacteriophage application. The extent of the reduction was dependent on the *S. enterica* strain, but the results do show benefits to using repeated bacteriophage applications during sprout germination to reduce *S. enterica* populations compared with a single bacteriophage application.

## 1. Introduction

Nontyphoidal *Salmonella enterica* (*S. enterica*) is one of the leading causes of laboratory-confirmed foodborne illness in the United States [1]. From 2014 to 2021, *Salmonella* was responsible for 57 out of 114 foodborne outbreaks in North America [2]. Within the 57 *Salmonella* outbreaks, 9 were of sprouts and 3 were specifically alfalfa sprouts [2]. Sprouts have been a concern for foodborne pathogens such as *Salmonella* because they are commonly consumed raw without any heat treatments [3]. Sprouts can be contaminated early as seeds in the food chain such as during the growth stage with contaminated irrigation water, harvest, processing, or the germination process where sprouting seeds can be soaked or rinsed in contaminated water [4,5,6,7]. During harvest and processing stage, contamination with *Salmonella* for sprout seeds could also happen through chance encounters with the fecal matter of animals, birds, and rodents [5]. The conditions during sprout seed germination are nutrient-rich and humid, which assist in promoting the growth of *Salmonella* already present on the seeds [8].

Conventionally, sprouts are recommended to be treated with chemical methods involving acetic acid, calcium hypochlorite, hydrogen peroxide, and lactic acid or physical treatments comprising heat, high pressure, or irradiation [8,9,10]. In Canada, it is recommended that a minimum three-log reduction in pathogens be achieved, but there is no legal requirement for seed sanitation [11]. The recommended calcium hypochlorite concentration of 20,000 ppm by the U.S. Food and Drug Administration (FDA) has been shown to completely eliminate *Salmonella* on alfalfa sprout seeds after a 10 min treatment [12]. However, 20,000 ppm calcium hypochlorite for 10 min or 15 min was unable to achieve complete *Salmonella* elimination on alfalfa sprout seeds in other studies [13,14]. The physical appearance of sprout seeds after chemical treatment could also be negatively affected. One study found 1000 and 2000 ppm calcium hypochlorite to cause inedible alfalfa sprout seed quality compared to tap water eight days after treatment [15]. Physical treatments such as heat, high pressure, and irradiation can be effective but can also negatively affect alfalfa seed germination depending on the parameters used [16,17,18]. Recent research combining physical and chemical treatments has used dry heat and 2% hydrogen peroxide or vacuumed hydrogen peroxide vapor with vacuumed dry heat to inactivate *Salmonella* Typhimurium on alfalfa seeds without negatively affecting germination [19,20]. Dry heat and 2% hydrogen peroxide can reduce *Salmonella* Typhimurium populations on alfalfa seeds by 1.66–3.60 log CFU/g, but long treatment times of up to 24 h may be undesirable [19]. Vacuumed dry heat at 73 °C and 30% vacuumed hydrogen peroxide vapor were shown to reduce *Salmonella* Typhimurium to <1 log CFU/g on alfalfa seeds; however, treatment times still required 120 min [20].

Even with the advances in seed sanitation research, these methods are unable to prevent pathogenic contamination that may be introduced during seed germination. Therefore, there has been interest in using natural biocontrol methods such as bacteriophages (phages). Phages are viruses first discovered in 1915 as a method to inactivate bacteria [21]. They are abundant and easily located in the ecosystem, which grants phages as an affordable antimicrobial agent [22]. Phages are naturally occurring, are highly specific, can self-replicate, and are generally nontoxic to humans [21,23]. Due to the self-replicating nature of phages, the concept is that phages can continually reduce pathogen populations introduced on sprouts by using the pathogen as hosts. Phages are flavorless, and their inclusion will not alter the sensory, textural, or nutritional properties of the food [23,24]. The high specificity of phages generally permits the native food microbiota to remain mostly unscathed, thus avoiding the inactivation of desirable microorganisms or undesirable effects from spoilage pattern modifications [21]. Sprouts are commonly perceived by consumers as organic, and the use of phages will align with the organic and natural benefits that sprouts provide [25]. Another benefit is that treatment with phages for alfalfa sprouts can be concurrently conducted with standard germinating procedures, thereby reducing treatment times that chemical and physical treatments may not provide.

Previous research has been conducted to investigate the effects of phages against *Salmonella* on sprouting seeds. A reduction of 1.37 logs in *Salmonella* growth was found with a single phage, Phage-A, in mustard sprout seeds in 24 h [26]. Another study used phage SSP6 against *S.* Oranienburg on alfalfa seeds at multiplicity of infection (MOI) 70 and found a reduction of 1 log CFU/g in *Salmonella* after 3 h of phage application, but the inhibitory effect did not last [27]. Phage SI1 was used against *S.* Enteritidis on sprouting alfalfa seeds at MOI 110, and there was a population reduction of ~2.5 log CFU/g immediately after phage treatment on day 1 [28]. However, by day 6, the population difference between control and phage-treated was <1 log CFU/g [28]. A higher population reduction of ~3.41 log CFU/g was achieved with a six-phage cocktail (F01, P01, P102, P700, P800, and FL41) against a *Salmonella* cocktail comprised of 11 serovars (Agona, Berta, Enteritidis, Hadar, Heidelberg, Javiana, Montevideo, Muenchen, Newport, Saint Paul, and Typhimurium DT104) on sprouting mung beans after 4 days of germination [29]. Similarly, another study used SalmoFresh, a six-phage cocktail, against a *Salmonella* cocktail composed of five strains (Newport, Braenderup, Typhimurium, Kentucky, and Heidelberg) on mung bean seeds at MOI 1000 [30]. A reduction of 1.83 log CFU/mL was found 1 h after phage treatment, but the reduction was reduced to 1.25 log CFU/mL after 72 h [30].

Previous studies have implemented one phage application against *Salmonella* on sprout seeds, and all have shown reduction; however, *Salmonella* population resurgence after phage treatment in the following days was common [27,28,30]. Therefore, the objective of this study was to determine the efficacy of repeated applications of phage during sprout seed germination on *S. enterica* populations compared with a single application.

## 2. Materials and Methods

### 2.1. Bacteriophage Propagation and Titer Measurement

Four *Salmonella* phages (SE14, SE20, SF5, and SF6) previously isolated in BC were used in the study [31]. The phage isolates were selected on the basis of their ability to lyse the *S. enterica* strains discussed in Section 2.2. Phage purification was performed according to the methods described by Fong et al. (2017) [28]. The *S. enterica* strains used for phage propagation are listed in Table 1. *S. enterica* cultures were prepared with overnight incubation at 37 °C in 10 mL Tryptic Soy Broth (TSB, Difco, Becton Dickinson, Franklin Lakes, NJ, USA) under agitation at 175 rpm. From the overnight cultures, 100 μL was inoculated into 10 mL of TSB and incubated at 37 °C under agitation at 175 rpm for 1.5 h until OD_600_ was measured to be between 0.2 and 0.4 with a UV-1800 UV/Vis Spectrometer (Shimadzu, MD, USA). One hundred microliters of 1.0 M CaCl_2_ (VWR International, Radnor, PA, USA) and 50 μL of phage (SE14, SE20, SF5, or SF6) were pipetted into the inoculum at OD_600_ 0.2–0.4 and incubated at 37 °C for 6 h. Afterward, the inoculum was poured into a 15 mL centrifuge tube (VWR International, Radnor, PA, USA) and sedimented at 4000× *g* for 10 min at 21 °C. The lysate was then poured into a separate centrifuge tube and filtered through a 0.4 μm filter (VWR International, Radnor, PA, USA). Phage titers were then measured by a plaque assay. The propagated phages were decimally diluted in 450 μL TSB + 1.0 mM CaCl_2_ (TSB-Ca) (VWR International, Radnor, PA, USA). Fifty microliters of *S. enterica* overnight culture was prepared according to the methods provided above, added to each decimal dilution, and incubated for 1 h at 37 °C under agitation at 175 rpm. Afterward, the contents were applied to the surface of tryptic soy agar (TSA, Difco, Becton Dickinson, Franklin Lakes, NJ, USA) + 1.0 mM CaCl_2_ (TSA-Ca). The plates were incubated at 37 °C for 24 h prior to counting the plaques. For 2.3, the phages were separated into 2 cocktails. Phage cocktail 1 comprised phages SE14, SE20, and SF6, and phage cocktail 2 comprised phages SE14, SF5, and SF6.

### 2.2. S. enterica Strains Storage Conditions and Preparation

Four *S. enterica* strains (*S.* Enteritidis S5-483, *S.* Newport S5-639, *S.* Muenchen S5-504, and *S.* Typhimurium S5-5336) were individually used in this study. All strains were maintained at −80 °C in TSB supplemented with 20% glycerol (VWR International, Radnor, PA, USA) for long-term storage. Working stocks were maintained in TSA and stored at 4 °C for a maximum of 4 weeks. For inoculation, *S. enterica* strains were individually prepared with overnight incubation at 37 °C in 10 mL TSB under agitation at 175 rpm for 18 h. The overnight cultures were then spun at 4000× *g* for 10 min, and the supernatant was decanted. The resulting pellets were washed twice with 10 mL, 0.1 M phosphate-buffered saline (PBS, VWR International, Radnor, PA, USA). The final suspension was diluted in 10 mL sdH_2_O for a concentration of ~10^8^ CFU/mL.

### 2.3. S. enterica and Bacteriophage Inoculation and Enumeration on Alfalfa Sprout Seeds

Alfalfa sprout seeds were obtained from a sprout grower in Saskatchewan, Canada. Twenty grams of sprout seeds was measured into each Whirl Pak bag (Nasco Whirl-Pak, Madison, WI, USA). Seeds were stored at 22 °C ± 1 °C until *S. enterica* inoculation where 2 mL of the *S. enterica* strain suspension was added to each Whirl Pak sample with alfalfa sprout seeds and vortexed for 2 min to achieve homogenous *S. enterica* inoculation in the seeds. All samples were air-dried in a biosafety cabinet for 1 h, resulting in a final *S. enterica* concentration of ~10^5^ CFU/mL. After 1 h, 15 mL of phage cocktail 1 or 2 was added to two-thirds of the seed samples for MOI 1000, and 15 mL of sdH_2_O was added to the other one-third. All samples were soaked for 2 h with gentle agitation at 175 rpm. After 2 h, the liquids were decanted, and 1 g of each sample was measured out into 5 mL microcentrifuge tubes (Simport Scientific, Saint-Mathieu-de-Beloeil, QC, Canada) and vortexed in 4 mL 0.1M PBS for 1 min. All seeds were stored in the dark at 22 ± 1 °C for 7 days. Each day, two-thirds of the sprouts were washed with 5 mL of sdH_2_O and one-third with 5 mL of phage cocktail 1 or 2 for treatments: control (no phage treatment, water soak + daily wash with sdH_2_O), single-phage treatment (phage soak + daily wash with sdH_2_O), and repeated-phage treatment (phage soak + daily phage washes). *S. enterica* populations were estimated on xylose lysine deoxycholate (XLD) agar (Oxoid, Nepean, ON, Canada) and TSA and total aerobic populations on TSA on days 0, 1, 3, and 7.

To determine if phage treatment had any impact on sprout yield, 20 g of alfalfa sprout seeds was measured into each Whirl Pak bag, individually inoculated with the *S. enterica* strains, and treated with no phage, a single phage, or repeated-phage treatments as described above. Sprouted seeds of all treatments were then weighed on day 7, and the results were statistically assessed as mentioned below in Section 2.4.

### 2.4. Statistical Analyses

Each treatment was conducted with four biological replicates. *S. enterica* populations were analyzed on log-transformed data with a one-way analysis of variance. The differences in alfalfa sprout seed weights between the three treatments were also assessed with a one-way analysis of variance. For means separation, Tukey’s honestly significant difference was performed. *p* values of <0.05 were considered as statistically significant. All statistical analysis was conducted with RStudio, version 1.1463 (RStudio, Inc., Boston, MA, USA).

## 3. Results and Discussion

### 3.1. S. enteritidis S5-483 Was the Most Susceptible to Phage Cocktail Treatments

**Figure 1:**
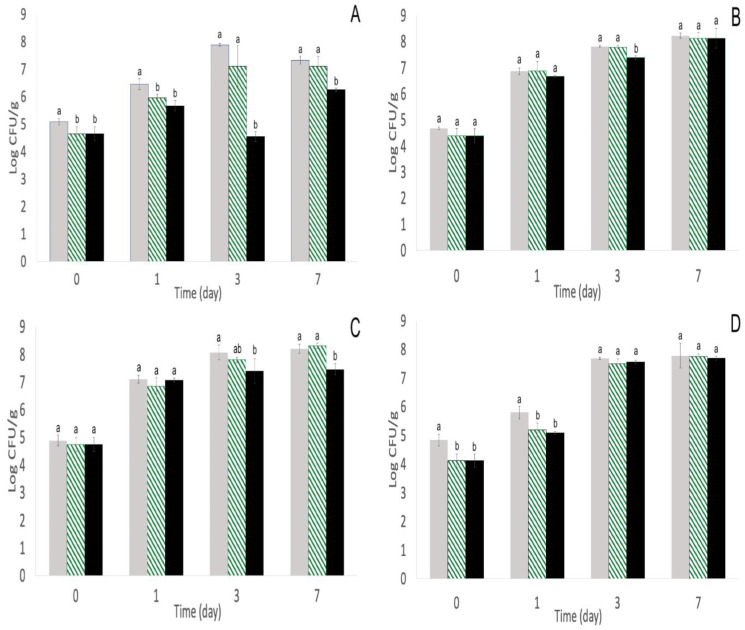
Populations of *S.* Enteritidis S5-483 (**A**), *S.* Muenchen S5-504 (**B**), *S.* Newport S5-639 (**C**), and *S.* Typhimurium S5-536 (**D**) after phage cocktail 1 (SE14, SE20, and SF6) infection on alfalfa sprout seeds estimated on XLD agar at 22 ± 1 °C on days 0, 1, 3, and 7. Treatments: 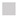 Control (no phage treatment, water soak and daily wash with sdH_2_O), 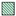 single-phage application (phage soak and daily wash with sdH_2_O), and 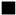 repeated-phage application (phage soak and daily phage washes). Different superscripts (a, b) each day indicate significant differences (*p* < 0.05) among treatments. Means and standard deviations were calculated using data from four biological replicates. Limit of detection > 0.2 log CFU/mL.

**Figure 2:**
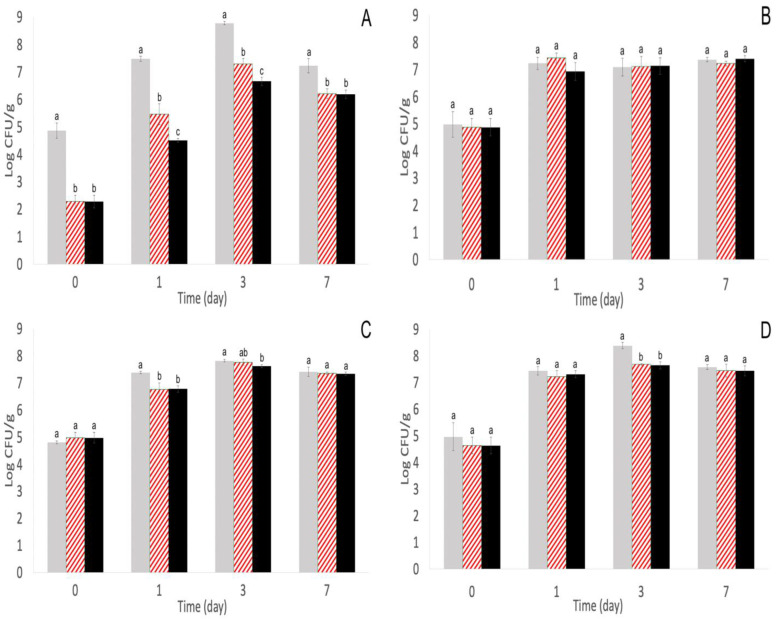
Populations of *S.* Enteritidis S5-483 (**A**), *S.* Muenchen S5-504 (**B**), *S.* Newport S5-639 (**C**), and *S.* Typhimurium S5-536 (**D**) after phage cocktail 2 (SE14, SF5, and SF6) infection on alfalfa sprout seeds estimated on XLD agar at 22 ± 1 °C on days 0, 1, 3, and 7. Treatments: 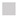 Control (no phage treatment, water soak and daily wash with sdH_2_O), 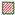 single-phage application (phage soak and daily water washes), and 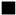 repeated-phage application (phage soak and daily phage washes). Different superscripts (a, b) for each day indicate significant differences (*p* < 0.05) among treatments. Means and standard deviations were calculated using data from four biological replicates. Limit of detection > 0.2 log CFU/mL.

From the four *S. enterica* strains inoculated onto alfalfa sprout seeds and treated with phage cocktails, *S.* Enteritidis S5-483 was the most susceptible to both phage cocktails on alfalfa sprouts. Phage cocktail 1 significantly (*p* < 0.05) reduced *S.* Enteritidis S5-483 populations by ~0.4 log CFU/mL on day 0, and repeated-phage treatments continued to significantly (*p* < 0.05) reduce populations with a further ~2.5 log CFU/mL reduction by day 3 (Figure 1A). By day 7, surviving *S.* Enteritidis S5-483 populations had a resurgence but were still ~0.9 log CFU/mL lower than single-phage and the control groups (Figure 1A). Phage cocktail 2 significantly (*p* < 0.05) reduced *S.* Enteritidis S5-483 populations by ~2.6 log CFU/mL on day 0, and repeated-phage treatments further reduced populations by ~0.7–1 log CFU/mL compared with a single-phage treatment and ~2.1–3 log CFU/mL compared with the control group on days 1 and 3 (Figure 2A). A single-phage treatment from phage cocktail 2 significantly (*p* < 0.05) reduced *S.* Enteritidis S5-483 populations by ~1.5–2 log CFU/mL on days 1 and 3, and by day 7, population reductions from both single- and repeated-phage treatments of phage cocktail 2 persisted and were 1 log CFU/mL lower than control (*p* < 0.05, Figure 2A).

In contrast, much smaller reductions of <0.8 log CFU/mL were observed in the other three strains (*S.* Newport S5-639, *S.* Muenchen S5-504, and *S.* Typhimurium S5-536) on various days treated with both cocktails (Figure 1B–D and Figure 2C,D). The exception was with *S.* Muenchen S5-504 and phage cocktail 2 because both single- and repeated-phage treatments did not significantly (*p* > 0.05) reduce *S.* Muenchen S5-504 populations compared with the control (Figure 2B). This outcome was unexpected because the phages were chosen for their ability to lyse each of the *S. enterica* strains. The exact reason for the decreased ability on sprouting seeds is unclear. Previous research indicated that phages in a cocktail have a greater efficacy than individual phages [32]. However, there is a chance that the usage of phages in a cocktail could result in antagonism where the efficacy of the cocktail to lyse specific *S. enterica* strains is decreased compared with individual phages [33]. One of the initial steps for phage–bacteria interaction is the binding of the phage to the receptors on the bacteria surface. On the *Salmonella* cell surface, vitamin B_12_ uptake outer membrane protein, flagellar, or lipopolysaccharide-related O-antigen proteins are all examples of receptors for phages [34]. OmpA and OmpC are examples of O-antigen proteins and have been essential for phage Sf6 infection in *Shigella* and *Salmonella* [35,36]. Similar key receptor sites were found on *E. coli* including Tsx and LamB [37]. Multiple phages in a cocktail could compete for the same host receptor sites, resulting in decreased cocktail efficacy compared with individual phage therapy [38].

### 3.2. Repeated-Phage Applications Further Decreased S. Enteritidis S5-483, S. Muenchen S5-504, and S. Newport S5-639 Populations

Even with the potential detriments of using a phage cocktail compared with single phages, it is still widely recommended because cocktails can prevent or decrease the emergence of phage-resistant bacteria compared with single-phage therapy [39]. For this study, repeated-phage applications did further decrease *S. enterica* populations depending on the strain, namely *S.* Enteritidis S5-483 from days 1, 3, and 7; *S.* Muenchen S5-504 on day 3; *S.* Newport S5-639 on day 7 with phage cocktail 1; and *S.* Enteritidis S5-483 on days 1 and 3 with phage cocktail 2 (Figure 1A–C and Figure 2A). With the implementation of phages, there is a concern that repeated applications will expand the community of phage-resistant bacteria. This could potentially be true for some strains such as *S.* Typhimurium S5-536 with phage cocktail 1 as populations were not significantly different (*p* < 0.05) on days 3 and 7 from control but was ~0.71 log CFU/mL lower compared with control on day 1 (Figure 1D). Conversely, for *S.* Newport S5-639 with phage cocktail 1, there was a ~0.75 log CFU/mL decrease on day 7 for repeated-phage application compared with control and single-phage application, but populations were not significantly different (*p* > 0.05) between treatments on days 0, 1, and 3 (Figure 1C). The population reduction on day 7 supports utilizing repeated-phage applications because this reduction may not have occurred with a single-phage application. The mechanism for the delayed phage infection is currently unclear and brings into question if the delay was due to the missed opportunity for the phage receptors to encounter the receptors on *S.* Newport S5-639 due to the immobility of phages or if there might be a mutation in the phage receptors that allowed infection on day 7 [40,41,42].

The population decrease from ~0.4 to 2.6 log CFU/mL on day 0 after a single-phage application was similar to the results from prior research (Figure 1A,D and Figure 2A). From [30], a six-phage cocktail at 10^8^ CFU/mL was used against a five-strain *Salmonella* mixture at 10^5^ CFU/mL. On mung bean sprouts, the phage cocktail reduced *Salmonella* populations by 0.83 log CFU/g with the spraying method and 2.16 log CFU/g with the immersing method but was not effective on mung bean seeds [30]. Similarly, another study displayed a 2.5 log CFU/g reduction of *S.* Enteritidis on sprouting alfalfa seeds from an initial concentration of 3.5 log CFU/g with an MOI of 110 PFU/CFU [28].

### 3.3. Estimation of S. enterica Populations on XLD and TSA Medias Shared the Same Trend despite Differences in Bacterial Counts

**Figure 3:**
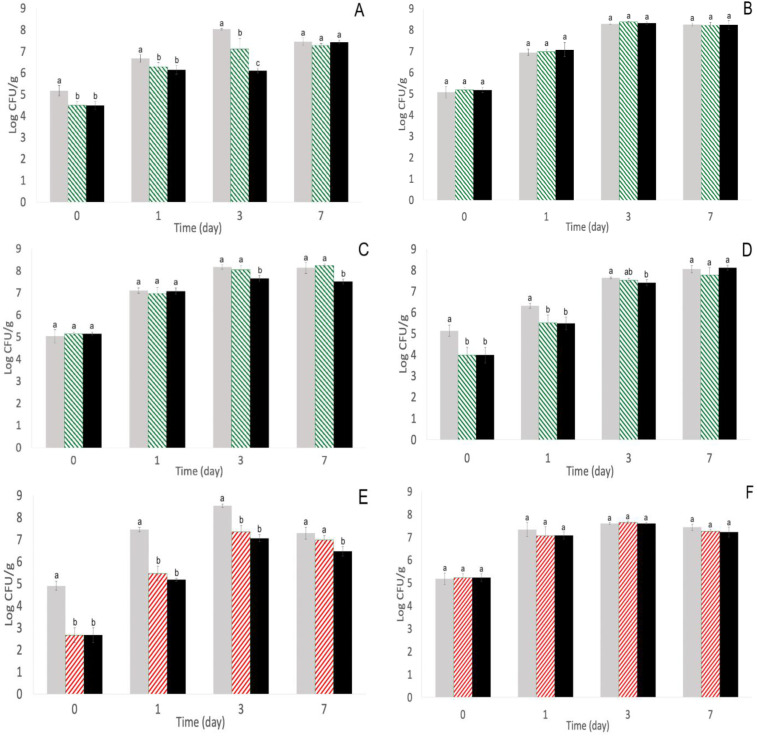

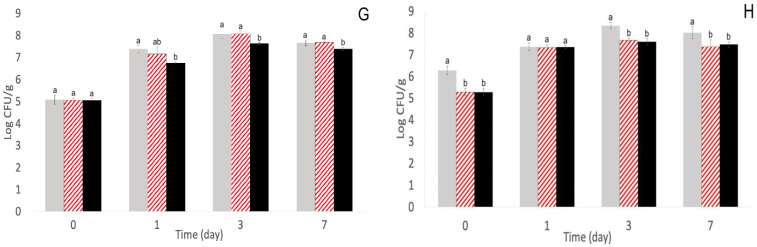
Populations of *S.* Enteritidis S5-483 (**A**), *S.* Muenchen S5-504 (**B**), *S.* Newport S5-639 (**C**), and *S.* Typhimurium S5-536 (**D**) after phage cocktail 1 (SE14, SE20, and SF6); and *S.* Enteritidis S5-483 (**E**), *S.* Muenchen S5-504 (**F**), *S.* Newport S5-639 (**G**), and *S.* Typhimurium S5-536 (**H**) after phage cocktail 2 (SE14, SF5, and SF6) infection on alfalfa sprout seeds estimated on TSA at 22 ± 1 °C on days 0, 1, 3, and 7. Treatments with phage cocktail 1: 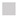 Control (no phage treatment, water soak and daily wash with sdH_2_O), 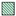 single-phage application (phage soak and daily wash with sdH_2_O), and 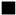 repeated-phage application (phage soak and daily phage washes). Treatments with phage cocktail 2: 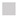 Control (no phage treatment, water soak and daily wash with sdH_2_O), 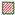 single-phage application (phage soak and daily water washes), and 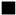 repeated-phage application (phage soak and daily phage washes). Different superscripts (a–c) each day indicate significant differences (*p* < 0.05) among treatments. Means and standard deviations were calculated using data from four biological replicates. Limit of detection > 0.2 log CFU/mL.

The population estimation of all four *S. enterica* strains on TSA against both phage cocktails (Figure 3) followed similar trends compared with the *S. enterica* populations estimated on XLD (Figure 1 and Figure 2), namely the significant (*p* < 0.05) decreases in *S.* Enteritidis S5-483 aerobic populations after single- and repeated-phage treatment for both phage cocktails (Figure 3A,E). Despite similar trends, the estimated populations on TSA were higher compared with XLD for certain strains and timepoints, namely *S.* Enteritidis S5-483 with phage cocktail 1 on day 3 after repeated-phage treatments and *S.* Typhimurium S5-536 with phage cocktail 2 on day 0 with control (Figure 1A, Figure 2D and Figure 3A,H). *S.* Enteritidis S5-483 populations with repeated-phage cocktail 1 treatment on day 3 were 4.55 ± 0.17 log CFU/mL on XLD, and the same treatment on TSA was 6.09 ± 0.11 log CFU/mL (Figure 1A and Figure 3A). The ~1.5 log CFU/mL difference might be attributed to the non-specificity of TSA. This difference was expected because populations estimated on TSA would comprise of both *S. enterica* and the microbiota present on the alfalfa sprout seeds, and the populations estimated on XLD were selective for *Salmonella*. *S.* Typhimurium S5-536 control populations with phage cocktail 2 on day 0 were 4.97 ± 0.52 log CFU/mL on XLD and 6.27 ± 0.20 log CFU/mL on TSA (Figure 2D and Figure 3H). The populations estimated on XLD on day 0 is very close to the *S. enterica* inoculum level of ~10^5^ CFU/mL; therefore, the difference in populations between the different media at the start of seed germination could indicate that the seeds used for *S.* Typhimurium S5-536 with phage cocktail 2 had a higher microbial population compared with the seeds used for other *S. enterica* strains and phage cocktails. The higher microbial population can be attributed to differences in harvesting, processing, or handling methods [4,5,6,7]. *S.* Typhimurium S5-536 populations on days 1 and 3 across all treatments were similar on both XLD and TSA, and this may support the idea that *S. enterica* growth between days 0 and 1 may have outcompeted other microbial populations on the sprouting seeds [43].

### 3.4. Phage Cocktail SE14, SF5, and SF6 Decreased Aerobic Populations on Alfalfa Sprout Seeds on Days 0 and 7

**Figure 4:**
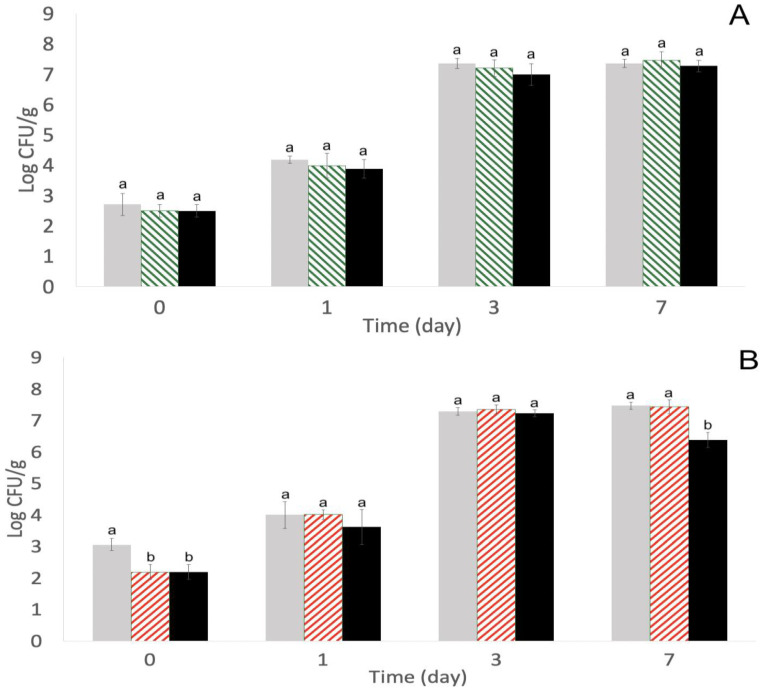
Total aerobic populations after phage cocktail 1 (SE14, SE20, and SF6) (**A**) and after phage cocktail 2 (SE14, SF5, and SF6) (**B**) infection on alfalfa sprout seeds estimated on TSA at 22 ± 1 °C on days 0, 1, 3, and 7. Treatments with phage cocktail 1: 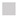 Control (no phage treatment, water soak and daily wash with sdH_2_O), 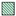 single-phage application (phage soak and daily wash with sdH_2_O), and 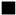 repeated-phage application (phage soak and daily phage washes). Treatments with phage cocktail 2: 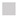 Control (no phage treatment, water soak and daily wash with sdH_2_O), 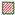 single-phage application (phage soak and daily water washes), and 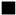 repeated-phage application (phage soak and daily phage washes). Different superscripts (a, b) each day indicate significant differences (*p* < 0.05) among treatments. Means and standard deviations were calculated using data from four biological replicates. Limit of detection > 0.2 log CFU/mL.

Alfalfa sprout seeds without *S. enterica* inoculation were separately inoculated with the two phage cocktails. With phage cocktail 1, there were no significant (*p* > 0.05) differences in aerobic microbial populations after phage treatments compared with control (Figure 4A). This is the trend that is expected because the *S. enterica* phages utilized are expected to only target *S. enterica* [44]. Conversely, there were significant (*p* < 0.05) differences with aerobic populations after the inoculation of phage cocktail 2 on days 0 and 7 with repeated-phage treatment, and the population decreases were 0.8 log CFU/mL and 1.1 log CFU/mL, respectively (Figure 4B). The phages used in this study were *S. enterica* phages, and the expectation was that they would only target *S. enterica* and leave the aerobic populations native to alfalfa sprouts undisturbed. Therefore, the aerobic population decreases with phage cocktail 2 on days 0 and 7 were unexpected. However, prior research found a phage, SH7, to lyse strains of *E. coli* 0157:H7, *Salmonella* Paratyphi, and *Shigella dysenteriae* [45]. Alfalfa sprout seeds are naturally abundant with microorganisms from a multitude of bacterial families, namely Enterobacteriacae, Acinetobacter, Janthinobacterium, and Pseudomonas [46]. *E. coli* 0157 and non-0157 and *Salmonella* are all representatives of the Enterobacteriacae family, and all have the potential to naturally be present on alfalfa sprout seeds [47,48]. There is a possibility for the phages in this study to infect distinct but related bacteria in the same family, but conclusions cannot be drawn without further genomic analyses. Despite the aerobic population decreases with phage cocktail 2, the final weights of the seeds with control (21.98 ± 0.66 g), single-phage treatment (21.76 ± 0.53g), and repeated-phage treatment (21.82 ± 0.45 g) were not significantly different (*p* > 0.05) from each other. This demonstrates that phage treatment does not affect the sprout yield, but further analyses into whether the aerobic population decreases inactivated desirable microorganisms or caused undesirable effects from spoilage pattern modifications will still need to be determined with this specific phage cocktail.

## 4. Conclusions

The results indicated that, on germinating alfalfa sprout seeds, two separate phage cocktails reduced *S. enterica* populations by ~0.4–3 log CFU/mL. The phage cocktails were unable to completely eliminate *S. enterica* populations, but repeated-phage applications were able to further reduce *S. enterica* populations compared with a single application from days 0 to 7. The reduction from repeated-phage applications was dependent on the strain, but the results do show advantages to using more than a single-phage application. To the best of our knowledge, there has not been any research conducted thus far with more than one phage application, and this study provided insight into the efficacy of repeated-phage applications against *S. enterica* during alfalfa sprout seed germination. Further research into alternating between different phage cocktails each day during sprout germination may be beneficial to further reduce *S. enterica* populations.

## Figures and Tables

**Table 1:** Bacteriophages used in this study.

Bacteriophage	*Salmonella* Host Strain for Propagation	Reference
SE14	Typhimurium S5-536	[31]
SE20	Muenchen S5-504	[31]
SF5	Enteritidis S5-483	[31]
SF6	Newport S5-639	[31]

## Data Availability

Not applicable.
